# Meta-analysis of short- and long-term outcomes after pure laparoscopic versus open liver surgery in hepatocellular carcinoma patients

**DOI:** 10.1007/s00464-018-6431-6

**Published:** 2018-09-10

**Authors:** Jan Witowski, Mateusz Rubinkiewicz, Magdalena Mizera, Michał Wysocki, Natalia Gajewska, Mateusz Sitkowski, Piotr Małczak, Piotr Major, Andrzej Budzyński, Michał Pędziwiatr

**Affiliations:** 1 2nd Department of General Surgery, Faculty of Medicine, Jagiellonian University Medical College, Kopernika 21 Street, 31-501 Krakow, Poland; 2 Center for Research, Training and Innovation in Surgery (CERTAIN Surgery), Krakow, Poland

**Keywords:** Laparoscopic liver resection, Hepatocellular carcinoma, Meta-analysis, Systematic review

## Abstract

**Background:**

The advantages of laparoscopy are widely known. Nevertheless, its legitimacy in liver surgery is often questioned because of the uncertain value associated with minimally invasive methods. Our main goal was to compare the outcomes of pure laparoscopic (LLR) and open liver resection (OLR) in patients with hepatocellular carcinoma.

**Methods:**

We searched EMBASE, MEDLINE, Web of Science, and The Cochrane Library databases to find eligible studies. The most recent search was performed on December 1, 2017. Studies were regarded as suitable if they reported morbidity in patients undergoing LLR versus OLR. Extracted data were pooled and subsequently used in a meta-analysis with a random-effects model. Clinical applicability of results was evaluated using predictive intervals. Review was reported following the PRISMA guidelines.

**Results:**

From 2085 articles, forty-three studies (*N* = 5100 patients) were included in the meta-analysis. Our findings showed that LLR had lower overall morbidity than OLR (15.59% vs. 29.88%, *p* < 0.001). Moreover, major morbidity was reduced in the LLR group (3.78% vs. 8.69%, *p* < 0.001). There were no differences between groups in terms of mortality (1.58% vs. 2.96%, *p* = 0.05) and both 3- and 5-year overall survival (68.97% vs. 68.12%, *p* = 0.41) and disease-free survival (46.57% vs. 44.84%, *p* = 0.46).

**Conclusions:**

The meta-analysis showed that LLR is beneficial in terms of overall morbidity and non-procedure-specific complications. That being said, these results are based on non-randomized trials. For these reasons, we are calling for randomization in upcoming studies. Systematic review registration: PROSPERO registration number CRD42018084576.

**Electronic supplementary material:**

The online version of this article (10.1007/s00464-018-6431-6) contains supplementary material, which is available to authorized users.

Laparoscopic liver resection is considered a feasible alternative to the open approach. Minimally invasive techniques have been widely used in the treatment of benign diseases such as hydatid cysts, hepatolithiasis, hemangiomas, focal nodular hyperplasia, and hepatic adenomas [1–3]. However, an increasing number of case series of malignant lesions scheduled for the laparoscopic approach have been published in the literature [4]. For instance, in other types of surgery laparoscopic access has been shown to be not inferior to an open approach in terms of oncological outcomes [5–7]. Most importantly, there are major well-known advantages related to the minimally invasive approach: less postoperative pain, lower morbidity, faster recovery, and better quality of life [8, 9].

Despite laparoscopy gaining popularity, its clinical utility and complexity in liver surgery, especially extensive liver resection, is still a subject of thorough analysis and discussion [10, 11]. It is often questioned whether reduced complications are enough to outweigh the benefits of open surgery such as the less steep learning curve and potentially shorter operative time. There is, however, evidence supporting non-inferior outcomes, primarily overall survival (OS) and disease-free survival (DFS).

Moreover, operations for hepatocellular carcinoma (HCC) in patients with liver cirrhosis and portal hypertension are considered difficult and may be associated with relatively high morbidity [12]. It has been proved that patients with liver cirrhosis have worse overall outcomes and a higher perioperative complication rate [13, 14].

So far only few meta-analyses comparing laparoscopic and open liver resections for HCC have been performed, and have not taken into consideration variances of the techniques such as pure laparoscopic and hand-assisted. This may create potential bias when drawing conclusions [15–18]. In addition, these reviews do not cover the many large-scale studies published in recent years.

To our best knowledge, this is the first systematic review and meta-analysis to evaluate exclusively pure laparoscopic liver resection (LLR) compared with open liver resection (OLR) for HCC.

The aim of this study was to evaluate different aspects of LLR, including its safety (morbidity and mortality), difficulty (operative time and blood loss), and clinical utility (long-term survival) in comparison with OLR.

## Materials and methods

### Search strategy

Our literature search included EMBASE, MEDLINE, Web of Science, and The Cochrane Library databases. The search terms used were “laparoscopy,” “pure laparoscopic,” “minimally invasive,” “liver resection,” “hepatectomy,” “hepatocellular carcinoma,” and their combinations with Boolean “AND” and “OR” operators. There were no date restrictions and only full texts in English were included. Our last search was performed on December 1, 2017. The full search strategy is available in Supplementary File 1. The systematic review was registered and its protocol published in the International Prospective Register of Systematic Reviews (PROSPERO) under registration number CRD42018084576.

### Study selection

Results of the initial search were screened independently by two teams with three reviewers in each team. Studies containing data comparing morbidity between patients undergoing pure laparoscopic and open liver resection for HCC were considered eligible for inclusion. All studies describing hand-assisted or hybrid resections (without subgroup data on pure laparoscopic resections), national registries, reviews, and animal studies were excluded. Both non-randomized and randomized studies were eligible as long as they matched the inclusion criteria.

### Data extraction and outcome measures

Outcomes of this systematic review were overall morbidity, major morbidity, specific complications (bile leak, abscesses, cardiopulmonary), blood loss, surgical site infection rate, conversion rate, operative time, reoperation and readmission rates, R0 resection rate, length of hospital stay, and 3- and 5-year OS and DFS rates. Data on type of study, number of patients enrolled, patients’ age and sex, tumor size, types of surgery, and liver function status (cirrhosis, Child scale) were also extracted. Major morbidity was extracted when stated or—if the Clavien–Dindo scale was used—complications rated as Clavien–Dindo grade 3 and higher were considered major.

### Statistical analysis

The analysis was performed using RevMan 5.3 (freeware from The Cochrane Collaboration) and R version 3.4.3 with meta package [19]. Statistical heterogeneity and inconsistency were measured using Cochran’s *Q* test and *I*^2^, respectively. Qualitative outcomes from individual studies were analyzed to assess individual and pooled risk ratios (RR) with pertinent 95% confidence intervals (CI) favoring pure laparoscopic over open liver resection for HCC and by means of the random-effects method. When appropriate, mean and standard deviation (SD) were calculated from medians and interquartile ranges using a method proposed by Hozo et al. [20]. Weighted mean differences with 95% CI are presented for quantitative variables using the inverse variance random-effects method. Statistical significance was observed at the two-tailed 0.05 level for hypothesis and 0.10 level for heterogeneity testing, while unadjusted *p* values were reported accordingly. To help with clinical interpretation of heterogeneity, we computed prediction intervals (PIs), as suggested by IntHout et al. [21], with the meta R package utilizing the approach of Higgins [22].

### Quality assessment

The quality of non-randomized studies was evaluated with the Newcastle–Ottawa Scale (NOS), which consists of three factors: patient selection, comparability of the study groups, and assessment of outcomes. A score ranging from 0 to 9 is assigned to each study, and those that achieve a score of 6 or greater are considered of high quality. The Cochrane risk of bias tool was used to assess the quality of the included randomized controlled trials. We used funnel plots and Egger’s test with meta-regression model to explore possible publication bias [23]. In cases of funnel plot asymmetry, the trim-and-fill method was applied to estimate the cause of asymmetry and correct it [24].

This review was performed strictly following Preferred Reporting Items for Systematic Reviews (PRISMA) guidelines [25] and the MOOSE consensus statement [26].

## Results

### Study identification

**Fig. 1:**
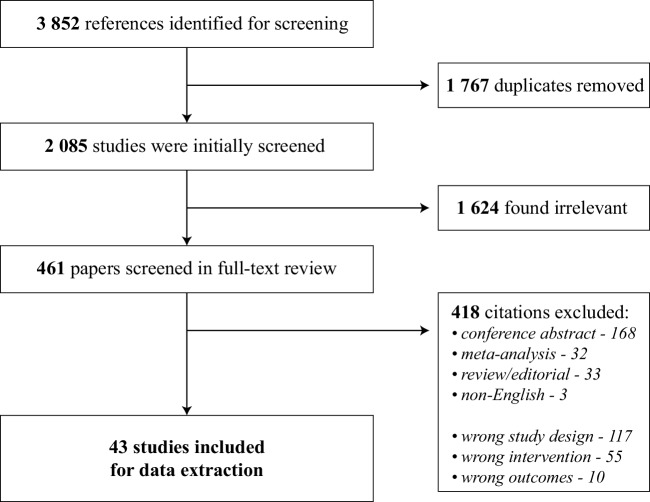
PRISMA flowchart of the study

The initial search yielded 3852 articles. After removing 1767 duplicates, 2085 studies were screened by their titles and abstracts for further analysis. Since 1624 did not match the review criteria, 461 full-text articles were screened for eligibility and of these, 418 were later excluded. The PRISMA flowchart and reasons for study exclusion are shown in Fig. 1.

### Characteristics of included studies

The characteristics of a total of 5100 patients from 43 studies included in the meta-analysis are specified in Table 1 [27–69]. The only randomized controlled trial was conducted by Jiang et al. [48].

**Table 1:** Included studies sorted by year descending

Study [Ref.]	Year	Country	No. of patients	Cirrhosis	Child–Pugh A/B/C	Tumor size	Major hepatectomies	Hospital volume	Readmissions	Pringle maneuver use	NOS quality score/Cochrane bias
			Total (lap vs. open)	% (lap vs. open)	Lap vs. open	cm (SD) (lap vs. open)	% (lap vs. open)	All resections for HCC/years		% (lap vs. open)	
Amato et al. [27]	2017	Italy	29 (11 vs. 18)	ND	100% A	ND	0% vs. 11.11%	ND	ND	ND	8
Chen et al. [28]	2017	China	257 (99 vs. 158)	ND	95/4 vs. 148/10	3.9 (1.53) vs. 4.0 (2.15)	0%	534/1.5	ND	ND	7
Guro et al. [29]	2017	Korea	104 (46 vs. 58)	58.70% vs. 65.52%	41/2/3 vs. 51/2/2	2.8 (1.4) vs. 4.7 (5.25)	17.39% vs. 31.03%	ND	ND	ND	8
Li et al. [30]	2017	China	220 (133 vs. 87)	ND	101/32 vs. 62/25	2.0 (0.5) vs. 2.3 (0.5)	ND	220/5.5	ND	ND	7
Tarantino et al. [31]	2017	Italy	64 (13 vs. 51)	100% vs. 96.08%	9 A vs. 46 A	ND	0%	ND	ND	0% vs. 23.53%	6
Xu et al. [32]	2017	China	64 (32 vs. 32)	100%	ND	4.3 (2.25) vs. 6.2 (1.23)	ND	336/2	ND	ND	8
Xu et al. [33]	2017	China	109 (50 vs. 59)	86% vs. 84.75%	44/6 vs. 53/6	3.38 (1.99) vs. 4.03 (2.67)	ND	109/5	0% vs. 3.39%	ND	7
Yoon et al. [34]	2017	Korea	66 (33 vs. 33)	100%	100% A	3.31 (1.65) vs. 2.96 (1.5)	ND	152/7	ND	96.97% vs. 93.94%	8
Ahn et al. [35]	2016	Korea	125 (32 vs. 93)	75% vs. 66.67%	28/2 vs. 83/9	3.1 (1.9) vs. 3.02 (2.3)	6.25% vs. 15.05%	137/13	ND	ND	7
Cheung et al. [36]	2016	China	440 (110 vs. 330)	100%	100% A	2.6 (1.57) vs. 2.85 (1.53)	10.00% vs. 12.42%	1,358/13	ND	ND	8
Harada et al. [37]	2016	Japan	68 (20 vs. 48)	ND	ND	2.4 (1.6) vs. 2.2 (0.7)	0% vs. 6.25%	88/7.5	ND	ND	8
Jiang et al. [38]	2016	China	118 (59 vs. 59)	ND	100% A	3 (0.75) vs. 3 (1.25)	ND	ND	ND	ND	8
Lai et al. [39]	2016	China	61 (28 vs. 33)	64.29% vs. 66.67%	ND	3.0 (1.1) vs. 3.3 (1.1)	ND	2,913/6	ND	ND	8
Sotiropoulos et al. [40]	2016	Greece	32 (11 vs. 21)	ND	ND	ND	0% vs. 23.81%	32/4.5	ND	18.18% vs. 71.43%	7
Sposito et al. [41]	2016	Italy	86 (43 vs. 43)	100%	42/1 vs. 41/2	ND	2.33% vs. 4.65%	271/8	ND	ND	8
Xiang et al. [42]	2016	China	335 (128 vs. 207)	81.25% vs. 80.68%	108/20 vs. 183/24	6.7 (1.5) vs. 6.9 (1.5)	ND	394/3	ND	41.41% vs. 41.55%	8
Zhang et al. [44]	2016	China	45 (20 vs. 25)	100%	100% A	ND	100%	ND	0%	ND	6
Zhang et al. [43]	2016	China	77 (35 vs. 42)	ND	ND	ND	100%	ND	ND	ND	6
Cho et al. [45]	2015	Korea	43 (24 vs. 19)	ND	ND	3.7 (1.8) vs. 4.8 (2.5)	ND	ND	ND	ND	8
Han et al. [46]	2015	Korea	176 (88 vs. 88)	62.50% vs. 59.09%	79/6/3 vs. 77/9/2	3.2 (2.07) vs. 3.5 (2.67)	30.68% vs. 26.14%	389/10	ND	29.55% vs. 14.77%	8
Harimoto et al. [47]	2015	Japan	65 (26 vs. 39)	100%	24/2 vs. 38/1	2.4 (1.6) vs. 2.2 (0.7)	ND	160/4	ND	ND	6
Jiang et al. [48]	2015	China	100 (50 vs. 50)	80% vs. 72%	ND	3.18 (0.29) vs. 3.22 (0.31)	ND	100/4.5	ND	ND	Low risk of bias
Lee et al. [49]	2015	Canada	129 (43 vs. 86)	41.86% vs. 38.37%	41/1/1 vs. 81/2/3	ND	ND	43/6.5	2.33% vs. 18.6%	ND	7
Luo et al. [50]	2015	China	106 (53 vs. 53)	ND	100% A	3.0 (0.75) vs. 3.0 (1.25)	0%	ND	ND	ND	7
Tanaka et al. [51]	2015	Japan	40 (20 vs. 20)	100%	100% A	2.33 (0.18) vs. 2.33 (0.23)	0%	592/7.5	ND	ND	8
Xiao et al. [52]	2015	China	127 (41 vs. 86)	80.49% vs. 83.72%	39/2 vs. 83/3	4.22 (2.05) vs. 4.30 (1.49)	14.63% vs. 12.79%	127/13	ND	ND	7
Yoon et al. [53]	2015	Korea	232 (58 vs. 174)	100%	53/5 vs. 158/16	2.87 (1.05) vs. 3.04 (1.18)	17.24% vs. 18.97%	1,050/4	ND	ND	9
Ahn et al. [54]	2014	Korea	102 (51 vs. 51)	68.63% vs. 66.67%	100% A	2.6 (1.5) vs. 2.8 (1.2)	3.92% vs. 5.88%	292/8	ND	ND	6
Kim et al. [55]	2014	Korea	146 (70 vs. 76)	ND	ND	2.58 (1.44) vs. 2.45 (1.27)	5.71% vs. 9.21%	ND	ND	0%	6
Memeo et al. [56]	2014	France	90 (45 vs. 45)	100%	44/1 vs. 43/2	3.2 (2.53) vs. 3.7 (3.73)	ND	332/19	ND	53.33% vs. 75.56%	8
Siniscalchi et al. [57]	2014	Italy	156 (23 vs. 133)	100%	ND	4.08 (1.53) vs. 3.6 (1.33)	13.04% vs. 6.02%	ND	ND	0%	6
Yamashita et al. [58]	2014	Japan	162 (63 vs. 99)	100%	59/4 vs. 96/3	ND	0%	653/14	ND	ND	9
Ai et al. [59]	2013	China	212 (75 vs. 137)	80.41% vs. 80.34%	59/38 vs. 107/74	7.85 (2.15) vs. 7.64 (2.36)	13.4% vs. 15.73%	275/4	ND	ND	7
Kobayashi et al. [60]	2013	Japan	51 (24 vs. 27)	ND	20/4 vs. 24/3	2.6 (1.1) vs. 2.2 (0.5)	0%	ND	ND	ND	7
Hu et al. [61]	2011	China	60 (30 vs. 30)	ND	29/1 vs. 24/6	6.7 (3.1) vs. 8.7 (2.3)	ND	60/5	ND	ND	6
Ker et al. [62]	2011	Taiwan	324 (116 vs. 208)	ND	98/17/1 vs. 197/10/1	2.5 (1.25) vs. 5.4 (3.5)	ND	324/8	ND	ND	6
Kim et al. [63]	2011	Korea	55 (26 vs. 29)	92.31% vs. 86.21%	ND	3.15 (1.75) vs. 3.6 (4.5)	19.23% vs. 24.14%	102/4.5	ND	0%	7
Lee et al. [64]	2011	Hong Kong	83 (33 vs. 50)	84.85% vs. 64%	100% A	2.5 (1.88) vs. 2.9 (1.95)	0%	233/6	ND	0%	7
Truant et al. [65]	2011	France	89 (36 vs. 53)	ND	32 A5 vs. 47 A5	2.9 (1.2) vs. 3.1 (1.2)	0%	122/7.5	ND	36.11% vs. 45.28%	9
Aldrighetti et al. [66]	2010	Italy	23 (11 vs. 12)	56.25% vs. 56.25%	9 A vs. 9 A	4 (2.2) vs. 4.6 (2.5)	0%	ND	ND	ND	8
Tranchart et al. [67]	2010	France	84 (42 vs. 42)	73.81% vs. 80.95%	30/1 vs. 33/1	3.58 (1.75) vs. 3.68 (2.09)	11.9% vs. 11.9%	156/9.5	ND	0% vs. 42.86%	8
Belli et al. [68]	2007	Italy	46 (23 vs. 23)	ND	100% A	3.1 (0.7) vs. 3.24 (0.7)	0%	106/4	ND	0% vs. 21.73%	9
Laurent et al. [69]	2003	France	27 (13 vs. 14)	ND	100% A	3.35 (0.89) vs. 3.43 (1.05)	0%	135/2	ND	100% vs. 85.71%	7

*ND* no data

#### Hospital volume

We estimated the volume of hospitals where studies were performed. Some institutes were noticeably very high-volume centers with almost 3000 cases in 6 years [39], while others performed as few as 60 procedures in 5 years [61].

#### Study quality

In all included studies, their quality was rated as high (≥ 6 by assessment using the NOS scale), and the risk of bias of the included randomized controlled trial was low according to Cochrane criteria.

#### Type of surgery

27 studies reported data on types of resections performed, including number of hemihepatectomies, although the reporting style and detail varied between articles.

#### Liver function

With respect to cirrhosis in patients, 27 studies reported data. Of these, 11 included only patients with cirrhosis. In total, 1065 out of 1257 (84.73%) and 1831 out of 2150 (85.16%) patients with cirrhosis were reported in LLR and OLR groups, respectively. Meanwhile, 33 studies reported data on patients’ Child–Pugh score, with 14 trials analyzing only subjects with Child–Pugh grade A.

#### Tumor size

35 manuscripts reported on tumor size. There is a noticeable trend of submitting patients with larger lesions to undergo OLR, leading to a potential yet incomputable bias. Pooled estimate analysis showed a significant trend toward smaller tumor sizes in LLR (mean difference − 0.26, 95% CI − 0.42 to − 0.10, *p* for effect < 0.001). However, the data are highly heterogeneous (*I*^2^ = 79%, *p* < 0.001).

#### Pringle maneuver

14 articles reported on use of the Pringle maneuver. 163 of 584 (27.91%) LLR patients and 250 of 865 (28.90%) OLR patients underwent this technique during surgery.

### Overall morbidity

**Fig. 2:**
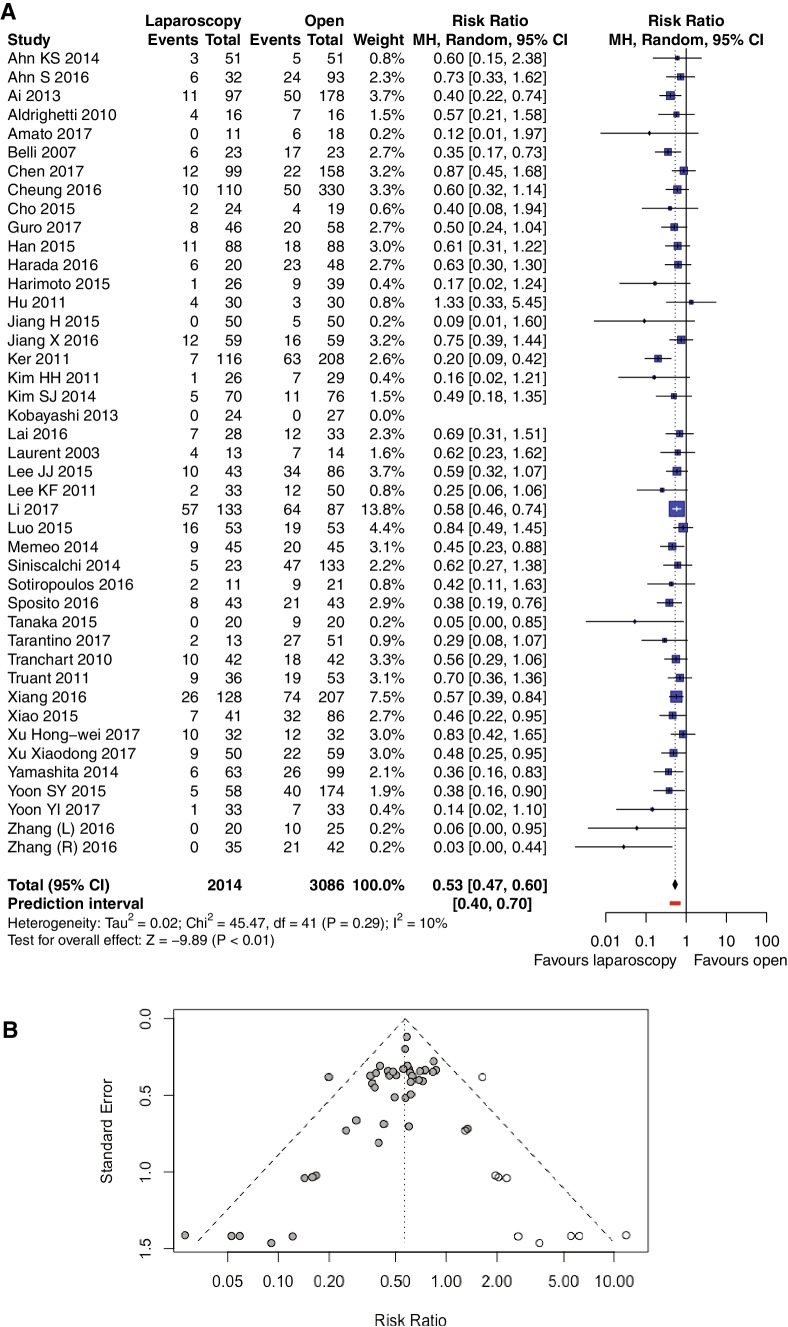
**A** Pooled estimates of overall morbidity for pure laparoscopic versus open liver resection for hepatocellular carcinoma. *CI* confidence interval, *df* degrees of freedom, *MH* Mantel–Haenszel. **B** Funnel plot for results from all studies after trim-and-fill analysis for overall morbidity. White dots represent filled-in studies

All studies reported on overall morbidity. The pooled analysis (Fig. 2A) indicates that LLR is connected with reduced overall morbidity (15.59%) compared with OLR (29.88%): RR = 0.53, 95% CI 0.47–0.60, *p* for overall effect < 0.00001, *p* for heterogeneity 0.29, *I*^2^ = 10%. The funnel plot is asymmetric and Egger’s regression test result significant, both of which indicate potential publication bias. Trim-and-fill analysis was performed and 10 studies that are mirror images of most outlying studies were filled in, [27, 34, 43, 44, 47, 48, 51, 62–64] as seen in Fig. 2B. Nevertheless, after this evaluation, the significance of the results remained unchanged, with RR = 0.57, 95% CI 0.49–0.65, and PI = 0.34–0.95.

### Major morbidity and mortality

**Fig. 3:**
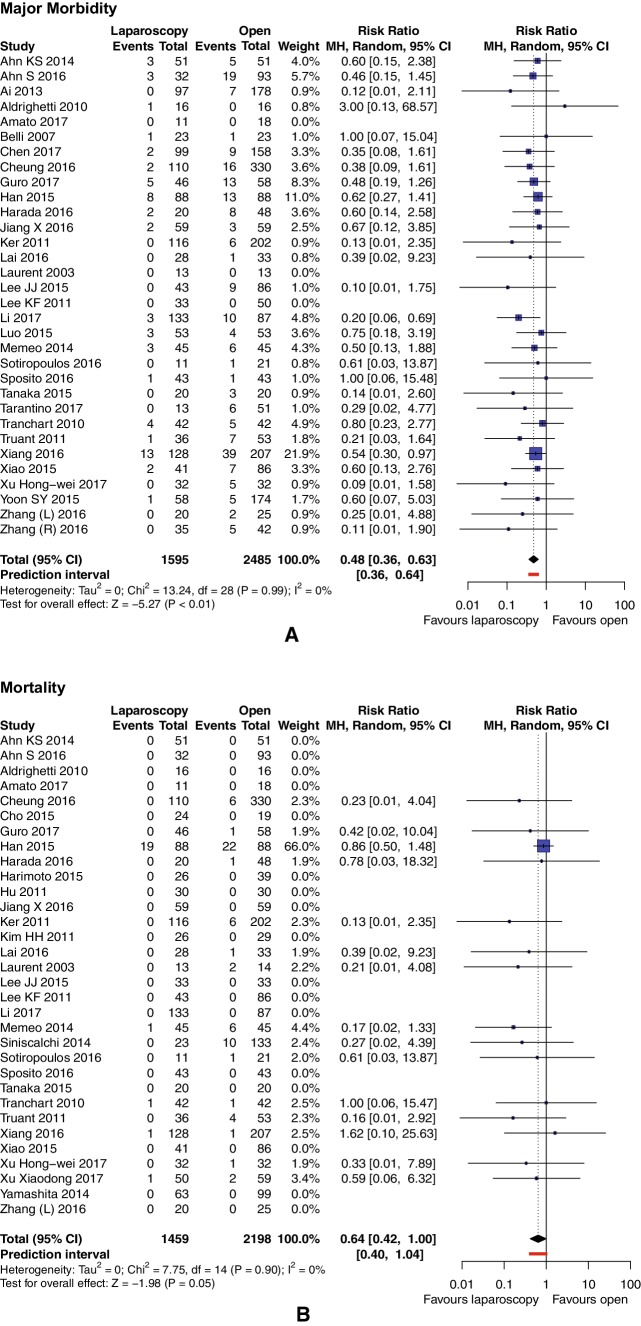
Pooled estimates of major morbidity (**A**) and mortality (**B**) for pure laparoscopic versus open liver resection for hepatocellular carcinoma. *CI* confidence interval, *df* degrees of freedom, *MH* Mantel–Haenszel

Major morbidity was reported in 32 studies (*n* = 4080 patients). Results in only two of them [30, 42] were significantly different, and pooled RRs favor LLR (3.76%) over OLR (8.69%), with RR = 0.48, 95% CI 0.36–0.63, *p* for effect < 0.00001, *p* for heterogeneity 0.99, *I*^2^ = 0%, and PI virtually equal to CI (Fig. 3A). Thirty-two studies (*n* = 3657 patients) reported data on mortality. Mortality rate in LLR was 1.58% versus 2.96% in OLR. Pooled analysis showed that mortality was also not significantly different between open and pure laparoscopic groups: RR = 0.64, 95% CI 0.42–1.00, *p* for effect 0.05, and *I*^2^ = 0%. PI was slightly wider than CI, being 0.40–1.04 (Fig. 3B).

### Complications

#### Bile leak

**Fig. 4:**
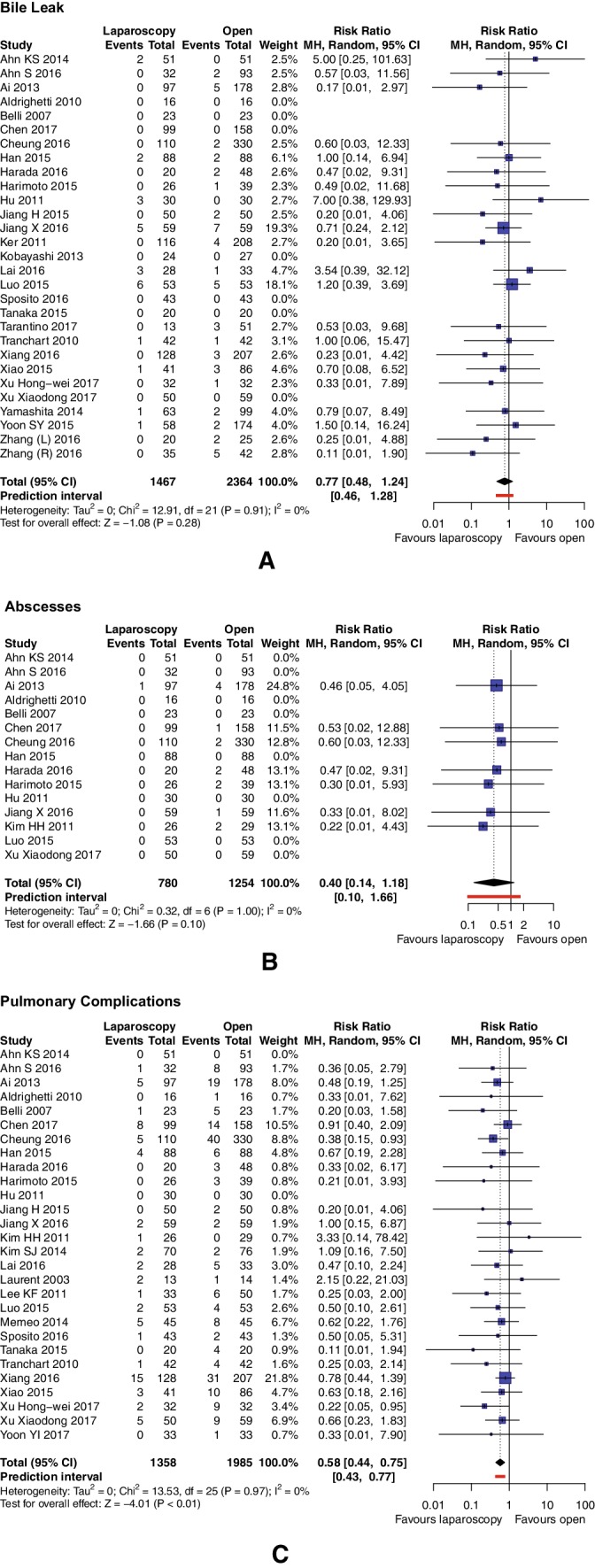
Pooled estimates of bile leak (**A**), abscesses (**B**), and pulmonary complications (**C**) for pure laparoscopic versus open liver resection for hepatocellular carcinoma. *CI* confidence interval, *df* degrees of freedom, *MH* Mantel–Haenszel

Bile leak rate was reported in 29 studies (*n* = 3831 patients). There were no significant differences between groups, with rates of 1.70% in the LLR group and 2.33% in the OLR group: RR = 0.77, 95% CI 0.48–1.24, *p* for effect 0.28, and *I*^2^ = 0% (Fig. 4A).

#### Abscesses

Fifteen trials (*n* = 2034 patients) reported on abscess occurrence. One out of 780 (0.13%) patients in the LLR group and 14 of 1254 (1.12%) in the OLR group had abscesses. Pooled analysis showed no statistical differences between these groups: RR = 0.40, 95% CI 0.14–1.18 *p* = 0.10, *I*^2^ = 0% (Fig. 4B).

#### Pulmonary complications

Twenty-eight studies (*n* = 3343 patients) reported the occurrence of pulmonary complications: 5.01% of patients undergoing LLR and 10.03% in the OLR had this morbidity, its rate being significantly reduced in the LLR group: RR = 0.58, 95% CI 0.44–0.75 (PI 0.43–0.77), *p* for effect < 0.0001, and *I*^2^ = 0% (Fig. 4C).

#### Blood loss

Data on blood loss were reported in 34 studies (*n* = 4116 patients). The heterogeneity for this outcome was very high, *I*^2^ = 94%. Sensitivity analysis did not find any potential sources of heterogeneity. For this reason, we decided not to pool the results.

#### Operative time

Operative time was reported in 43 studies (*n* = 5100 patients). We did not pool the results because of the very high heterogeneity (*p* < 0.0001, *I*^2^ = 91%). Sensitivity analysis did not find specific studies that caused these results.

#### Length of hospital stay

Length of hospital stay was reported in 42 studies (*n* = 5032 patients). However, heterogeneity was high (*I*^2^ = 85%) and its source was not revealed by sensitivity analysis. Thus, no pooling was performed.

#### Survival

**Fig. 5:**
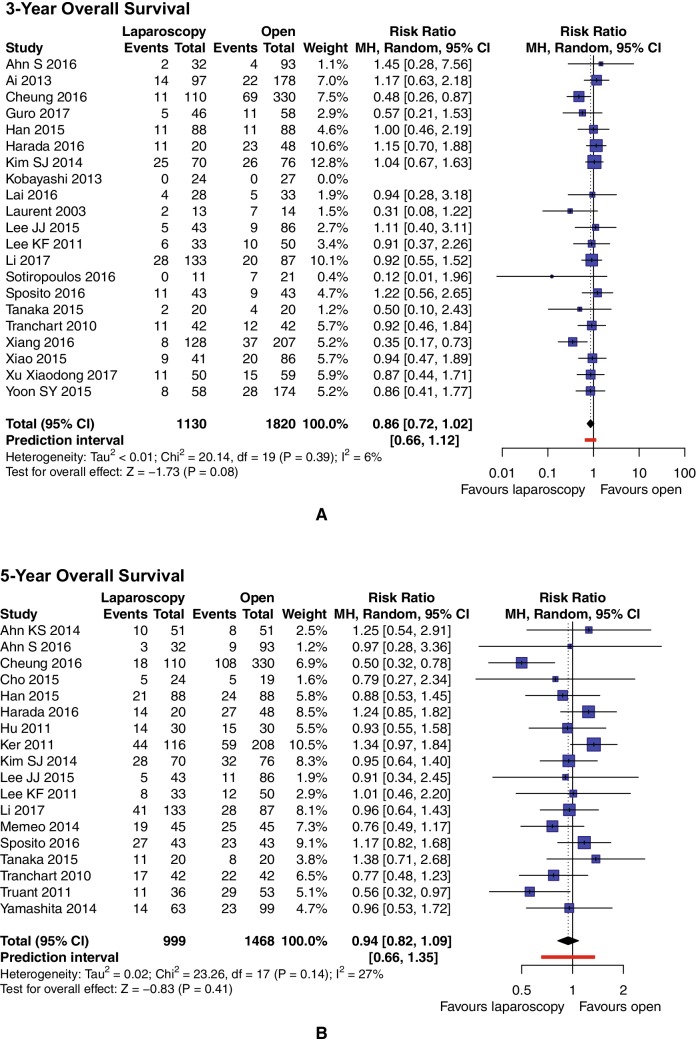
Pooled estimates of 3-year (**A**) and 5-year (**B**) overall survival for pure laparoscopic vs. open liver resection for hepatocellular carcinoma. *CI* confidence interval, *df* degrees of freedom, *MH* Mantel–Haenszel

Three-year OS was reported in 21 studies (*n* = 2950 patients), while 18 (*n* = 2467 patients) trials reported 5-year OS. There were no significant variations among the analyzed groups: the LLR group had OS rates of 83.72% and 68.97% in 3 and 5 years, respectively. The OS rate in patients undergoing OLR was 80.82% in 3 years and 68.12% in 5 years. For 3-year OS, RR = 0.86, 95% CI 0.72–1.02, *p* = 0.08, and *I*^2^ = 6% (Fig. 5A). For 5-year OS, RR = 0.94, 95% CI 0.82–1.09, *p* = 0.41, and *I*^2^ = 27% (Fig. 5B).

**Fig. 6:**
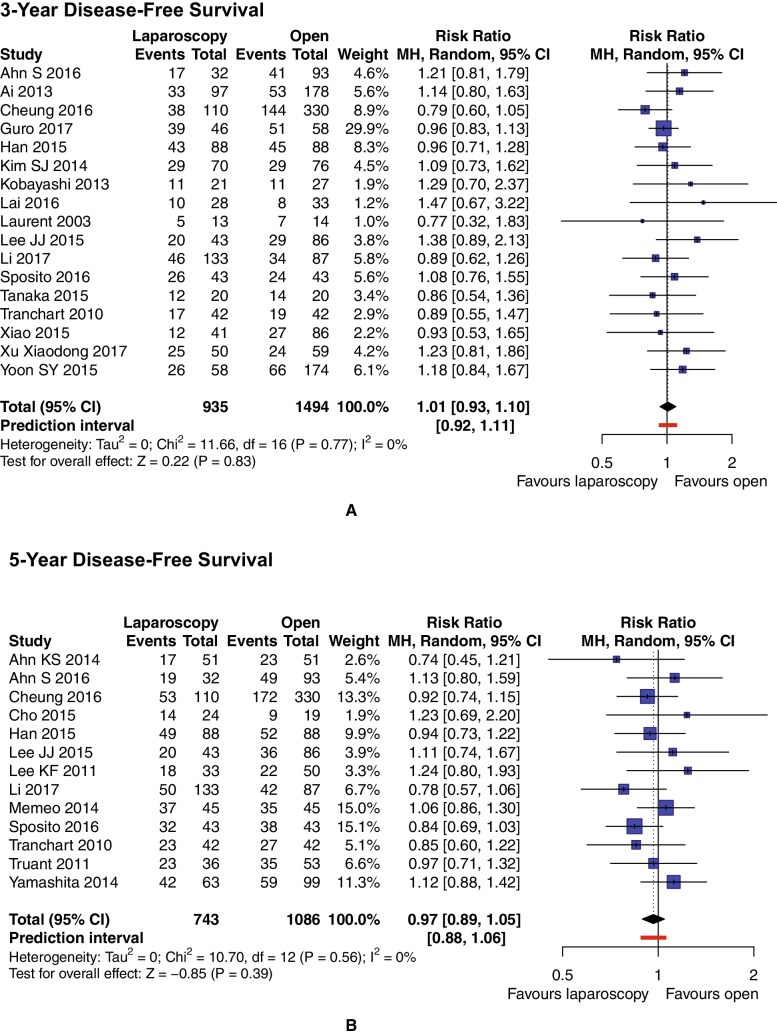
Pooled estimates of 3-year (**A**) and 5-year (**B**) disease-free survival for pure laparoscopic versus open liver resection for hepatocellular carcinoma. Forest plot presents studies after sensitivity analysis. *CI* confidence interval, *df* degrees of freedom, *MH* Mantel–Haenszel

Nineteen studies (*n* = 2836 patients) reported on 3-year DFS. Thirteen articles (*n* = 1829 patients) provided information on 5-year DFS. Pooled analysis showed no differences between groups for either outcome. The 3-year DFS rate was 59.45% in the LLR group and 59.05% in the OLR group, while the 5-year DFS rate was 46.57% for LLR patients and 44.84% for OLR patients. For 3-year DFS, RR = 1.02, 95% CI 0.90–1.15, and *p* = 0.81. We analyzed sources of moderate heterogeneity (*I*^2^ = 47%), and in sensitivity analysis we found two studies that affected the results [42, 64]. After their removal from the meta-analysis, pooling confirmed previous findings, with RR = 1.01 (95% CI 0.93–1.10) and virtually no heterogeneity (*I*^2^ = 0%) (Fig. 6A). For 5-year DFS, RR = 0.97, 95% CI 0.90–1.05, *p* = 0.46, and *I*^2^ = 0% (Fig. 6B).

## Discussion

### Summary of findings

There is growing evidence supporting the feasibility of laparoscopic liver resection for HCC, and its safety is confirmed in our meta-analysis. This review, including over 5000 patients, shows that pure laparoscopy significantly reduces morbidity while at the same time delivering survival comparable with that of open surgery. Because of the very high heterogeneity, it is not possible to definitively assess the differences in blood loss and operative time. Although the quality of all included studies was assessed using standardized tools as high, all but one are non-randomized, which may introduce selection bias. Moreover, there were differences in tumor size and in the use of the Pringle maneuver between LLR and OLR groups, which may cause serious bias and troublesome interpretation of results.

In addition, we did not include three studies because of the language limitations. However, based on abstract screening, number of cases in them was relatively small (less than 50 cases). Therefore, it is very unlikely that their inclusion would alter the final results.

We realize that our review is not the first to be conducted on this topic. However, previously published meta-analyses on liver resections for HCC either did not take the type of laparoscopic technique into consideration [16–18] or performed a subgroup analysis that mistakenly assigned trials with hybrid resections to the LRR group as in Sotiropoulos et al. [15]. This might have introduced a major methodological bias to previous studies. In addition, more recent large-scale trials are not included in previous reviews. Moreover, several meta-analyses, including recently published one by Goh et al., were limited to cirrhotic patients, which does not allow to draw conclusions with wide clinical applicability of laparoscopy [70].

These facts prompted us to revisit this topic and follow strict methodological and surgical criteria to obtain the best available evidence. Additionally, we used PIs to interpret whether the results would be applicable in different clinical settings.

When defining the aim of our study, we strived to tackle the issue on different levels in terms of LLR safety (by analyzing morbidity, mortality, and specific complications), difficulty (operative time and blood loss), and its long-term results (OS and DFS).

### LLR safety

Overall morbidity is crucial in our review for assessment of the method’s safety. Pooled analysis confirmed the benefits of laparoscopy with low heterogeneity and its demonstrable effect in different settings. It is worth noting that only one study, by Hu et al. [61], reported higher overall morbidity in LLR. Also, common general (i.e., pulmonary) complications were less likely to occur in the LLR patients, while procedure-associated complications (bile leak, abscesses) did not differ compared with OLR.

Nevertheless, results varied between studies: some showed an overall complication rate as high as 20.31% for LLR [42] while others reported no morbidity at all among 50 patients undergoing LLR [48]. Such discrepancies can be explained to some extent by hospital volume or surgeon experience, as discussed further in the limitations of our review below, but it seems that the definitions and reporting of specific complications are not standardized, which may result in significant discrepancies between included studies and eventual bias.

Pooled analysis also showed no differences in mortality (1.58% in LLR vs. 2.96% in OLR; RR = 0.64; 95% CI 0.42–1.00), which generally is a relatively rare complication in liver resections for HCC. Only one study by Xiang et al. [42] had a higher mortality rate in the LLR group, but it is important to note that this was based on one death in both groups: 1 out of 128 in LLR and 1 out of 208 in OLR.

### Difficulty of LLR and OLR

Laparoscopy in surgery is sometimes disparaged as being more complex, supposedly because of the steep learning curve, longer operation times, and greater blood loss [71]. However, it has been shown that more experienced surgeons have, in fact, shorter median operative times as well as reduced blood loss and conversion rates in liver surgery [72]. Many parameters, such as hospital volume, are difficult to compare, which directly affects the experience and subsequent intraoperative results. Some studies point out that an increase in operative time may be a result of the learning curve and should improve in the future [69]. Others, however, claim to have lowered it, as evidenced by a reduced conversion rate [36, 49]. This learning curve effect is nearly impossible to include in an analysis. These study limitations may be one reason why the legitimacy of LLR is often challenged in liver surgery. In our meta-analysis, we decided not to perform a pooled analysis of operative time, blood loss, and length of stay for reasons of significant heterogeneity. Even if pooling was possible, there is a potential bias because of highly variable operative techniques between centers. Most studies did not thoroughly describe the type of surgical devices and techniques of parenchyma transection, which influences the total blood loss. Another difference is the rate of use of the Pringle maneuver.

Usually laparoscopy is also associated with extended duration of surgery, but there is a possibility of patient selection bias reflecting surgeons’ preference to submit more complex cases to OLR. However, recent analyses showed that in liver resections a trend toward shorter operative time in laparoscopy may in fact be non-significant [73, 74]. Types of surgical instruments used in LLR may also affect the operative time [75].

The very high heterogeneity of these results (*I*^2^ = 94% for blood loss, *I*^2^ = 91% for operative time, and *I*^2^ = 85% for length of stay) does not allow us to draw definitive conclusions, and it would seem that non-randomized trials may not be able to resolve this issue.

### Long-term results

The meta-analysis confirmed previous findings [17, 18] that LLR does not differ from OLR in terms of OS and DFS. This has to be juxtaposed with the clear benefits of LLR safety as well as its vague yet potentially greater difficulty. Our meta-analysis of more than 5000 cases points out the weakness of non-randomized trials that do not allow for unequivocal conclusions. All studies but one, by Jiang and Cao [48], presented non-randomized groups. This, in our opinion, is a massive drawback that must be taken into account when discussing the data. We interpret our results as a plea for well-designed multicenter trials analyzing the type of surgery, complexity of the procedure, surgeon’s experience, and hospital volume.

Due to inconsistent reporting in included manuscripts, we did not analyze recurrence-free survival separately from DFS. Although a few publications did indeed evaluate recurrences in LLR versus OLR, mean follow-up time varies significantly between studies, making it impossible to pool results without bias.

A very important bias results from patient allocation. There are indisputable differences between LLR and OLR patients regarding the tumor size, cirrhosis, or, intraoperatively, use of the Pringle maneuver. This limitation can only be overcome by randomized controlled trials. Moreover, center volumes highly differ, as does surgeons’ experience. The latter was not reported in our included studies despite it being an extremely important factor in complex liver surgeries. In addition, use of the Pringle maneuver reported in the studies varies and resulted in very high heterogeneity. Finally, we did not analyze the potential impact of surgical technique and its differences between studies. Neither were perioperative care protocols considered, despite their application having been shown to be beneficial in many surgical disciplines [76, 77].

## Conclusions

This systematic review with a meta-analysis, thus far the most comprehensive analysis comparing pure LLR with OLR for HCC, reveals major flaws in the available literature. The results indicate that LLR is safe in different clinical settings as it may be associated with reduced overall morbidity and non-procedure-specific complications, and no negative influence on mortality as well as OS and DFS. However, these results are based on non-randomized trials comparing heterogeneous groups of patients, thus introducing confounding variables from the outset.

In our opinion, therefore, there is no need for further non-randomized trials proving the feasibility and safety of LLR. This is a plea for large, multicenter, well-designed randomized controlled trials that can overcome the weaknesses of the available evidence.

## Electronic supplementary material

Below is the link to the electronic supplementary material.

Supplementary material 1 (DOCX 12 KB)
