# Supplementary material for: Meta-analysis of short- and long-term outcomes after pure laparoscopic versus open liver surgery in hepatocellular carcinoma patients
Source: Surg Endosc. 2018 Sep 10;33(5):1491–507. doi: 10.1007/s00464-018-6431-6 (PMC6484823; doi:10.1007/s00464-018-6431-6)
Supplement: Supplementary file 1 — Supplementary material 1 (DOCX 12 KB) [file 464_2018_6431_MOESM1_ESM.docx]

**Supplementary File 1.**

**Full search strategy in Ovid database**

| Searches | Results |
| --- | --- |
| (hepatic resect* or liver resect* or hepar or hepatectom* or hemihepatect* or segmentectom* or bisegmentectom* or trisegmentectom*).ab,kw,ti. | 43 476 |
| (laparoscop* or minimal* invasiv* surger*).ab,kw,ti. | 164 380 |
| 1 and 3 | 37 530 |
| 2 and 4 | 1 357 |
